# Analysis of risk factors for hypoproteinemia after total hip arthroplasty and construction of a nomogram: A retrospective study

**DOI:** 10.1097/MD.0000000000047345

**Published:** 2026-01-30

**Authors:** Zhongyou Yu, Yongshuo Li, Yilv Zhang, Nanhai Qiu, Wengang Zhu

**Affiliations:** a Guangdong Medical University, Zhanjiang, China; b Yuebei People’s Hospital Affiliated to the Medical College of Shantou University, Shaoguan, Guangdong Province, China.

**Keywords:** postoperative hypoalbuminemia, prediction model, risk factors, total hip arthroplasty

## Abstract

Postoperative hypoalbuminemia (POHA) remains one of the major complications following total hip arthroplasty (THA) in patients with end-stasge hip disease. Identifying factors that can reduce the incidence of POHA is crucial for improving clinical outcomes in these patients. Our study aimed to develop and validate a nomogram that can pre-operatively quantify an individual patient’s risk of POHA. We retrospectively reviewed patients aged ≥65 years who underwent primary unilateral THA between January 2021 and December 2022. Inclusion criteria were: age ≥ 65 years, primary unilateral THA, pre-operative serum albumin > 30 g/L, and no history of liver disease, nephrotic syndrome or malignancy that could affect albumin metabolism. Exclusion criteria were: revision or secondary THA, incomplete medical records, or missing key peri-operative data. The primary outcome was the occurrence of POHA, defined as serum albumin < 30 g/L. Univariate and multivariate regression analyses were performed to identify independent risk factors for POHA, followed by the development of a nomogram. The discriminative ability, predictive accuracy, and clinical utility of the nomogram were evaluated using the area under the curve, calibration curves, and decision curve analysis. Among the 419 THA patients, POHA occurred in 130 cases (16.9%). Multivariate regression analysis revealed that female sex (odds ratio [OR] = 2.74, 95% confidence interval [CI]: 1.31–5.72, *P* = .007), longer operative time (OR = 1.02, 95% CI: 1.01–1.03, *P* = .006), elevated erythrocyte sedimentation rate (OR = 1.03, 95% CI: 1.01–1.05, *P* < .001), lower preoperative serum albumin (OR = 0.80, 95% CI: 0.72–0.88, *P* < .001), and higher alkaline phosphatase levels (OR = 1.01, 95% CI: 1.01–1.02, *P* = .045) were independent risk factors for POHA. A nomogram prediction model was constructed based on these 5 variables. The area under the curve values for the training and validation cohorts were 0.81 (95% CI: 0.74–0.87) and 0.76 (95% CI: 0.65–0.88). Female, prolonged operative time, elevated erythrocyte sedimentation rate, and higher alkaline phosphatase levels were identified as risk factors for POHA after THA, while higher preoperative serum albumin was a protective factor. Orthopedic surgeons should carefully consider POHA and its influencing factors when optimizing perioperative THA management.

## 1. Introduction

With increasing life expectancy and accelerated population aging, hip disorders have become a critical public health concern.^[1]^ Total hip arthroplasty (THA) is indicated for various conditions, including osteonecrosis of the femoral head, hip fractures, sequelae of septic or neoplastic arthritis after infection control, and other end-stage hip pathologies.^[2]^ Globally, hip fractures alone account for over 1.6 million cases annually, with a steadily rising incidence.^[3]^ Compared to internal fixation, THA is increasingly favored by clinicians due to its lower complication rates,^[4]^ reduced intraoperative blood loss,^[5]^ and decreased need for revision surgery.^[6]^

Postoperative hypoalbuminemia (POHA) is a major complication following THA. According to Wu et al, its incidence after primary THA reaches 37.7%.^[7]^ POHA can lead to severe postoperative sequelae, including pneumonia,^[8]^ acute kidney injury,^[9]^ surgical site infections, prolonged hospitalization, elevated mortality risk, and increased financial burdens for both patients and healthcare systems.^[10]^ Consequently, developing effective strategies to predict and prevent POHA has emerged as an urgent clinical priority, carrying significant theoretical and practical implications.

Current research in this field remains limited, and no consensus exists on the risk factors for POHA. Nomograms, which visually quantify the risk of specific outcomes, are widely used for disease diagnosis and prognosis prediction. This study aims to identify risk factors for POHA after THA and construct a nomogram model to assist clinicians in early risk stratification, optimized surgical planning, and targeted interventions, ultimately reducing POHA incidence.

## 2. Materials and methods

This study retrospectively analyzed complete clinical data from patients who underwent THA at Yuebei People’s Hospital of Shaoguan City between January 2021 and December 2022.

The study was conducted in accordance with the Declaration of Helsinki and approved by the Institutional Review Board of Yuebei People’s Hospital (No. YBSKY-2024-183-001). As a retrospective study, the ethics committee waived the requirement for individual patient informed consent.

Currently, no international gold standard exists for diagnosing POHA, likely due to significant variations in surgical approaches across different systems that complicate research efforts. The primary debate centers on whether the diagnostic threshold should be set at <30 g/L or <35 g/L. Clinically, serum albumin levels below 35 g/L are generally considered diagnostic for hypoalbuminemia (HA), consistent with findings by Brock et al^[11]^ The Common Terminology Criteria for Adverse Events (CTCAE) version 5.0 classifies serum albumin levels below 35 g/L as mild HA (typically requiring monitoring but not treatment), while levels between 20 and 30 g/L indicate moderate HA necessitating intervention, and lower levels demand urgent treatment. The European Society for Clinical Nutrition and Metabolism also recognizes serum albumin < 30 g/L as directly reflecting disease severity and nutritional status.^[12]^ Based on these studies and clinical relevance, this study defined POHA as postoperative minimum serum albumin levels < 30 g/L.

### 2.1. Selection criteria

Inclusion criteria: age ≥ 65 years; underwent primary unilateral THA; preoperative serum albumin > 30 g/L; and no history of liver disease, nephrotic syndrome, or malignancy that could affect albumin metabolism. Exclusion criteria: age < 65 years; revision or secondary hip replacement; and incomplete medical records or missing key perioperative data. Follow-up duration was not used as a selection criterion, because the primary outcome for POHA was captured within few days postoperatively via routine blood tests.

### 2.2. Data collection

Based on a comprehensive review of existing literature and multidisciplinary discussions, the following patient demographics, clinical characteristics, and perioperative parameters were systematically collected: sex, age (with elderly defined as ≥65 years), height, weight, osteoporosis (*T*-score ≤ −2.5 by dual-energy x-ray absorptiometry), hypertension history, diabetes history, chronic gastritis history, chronic obstructive pulmonary disease history, serum albumin, globulin, hemoglobin, white blood cell count, erythrocyte sedimentation rate (ESR), C-reactive protein (CRP), total bilirubin, uric acid, serum calcium, serum sodium, alkaline phosphatase, triglycerides, low-density lipoprotein, high-density lipoprotein, surgical approach (posterolateral approach and direct anterior approach), anesthesia method (combined spinal-epidural anesthesia and general endotracheal anesthesia), operation time, incision length, intraoperative blood loss, drainage tube placement (yes/no), drainage volume. The data are all within 1 week after THA.

### 2.3. Statistical analysis

Continuous variables are reported as mean ± SD or median [P25, P75], depending on normality, and compared using *t*-tests or Mann–Whitney *U* tests, respectively. Categorical variables are summarized as n (%) and compared using the χ² test. The analysis first justified that 53 POHA cases for 5 covariates satisfied the 10 to 20 events-per-variable rule.^[13]^ The cohort was randomly divided into training (n = 293) and validation (n = 126) sets. Univariate logistic regression was first applied to screen potential predictors (*P* < .05). Significant variables were then entered into a multivariate logistic regression model using backward stepwise selection (*P* < .05) to identify independent risk factors for POHA.

Discrimination was quantified by the area under the curve (AUC). Calibration was assessed with Hosmer–Lemeshow goodness-of-fit test and calibration plots. Clinical utility was evaluated by decision curve analysis. The analytical approach employed IBM SPSS Statistics software, Version 26 (IBM Corp., Armonk) for statistical tests and R 4.3.1 for modeling; multicollinearity was assessed with tolerance < 0.1 or variance inflation factor > 5.^[14]^

## 3. Results

### 3.1. Basic information in patient information

The study cohort comprised 419 patients with 57.35% aged ≥65 years (49.65% male, 50.35% female), demonstrating an overall POHA incidence of 16.90%. Patients were divided into training (n = 293, 18.00% POHA) and validation (n = 126, 14.20% POHA) groups, with Table 1 showing no significant intergroup differences (*P* > .05, α < 0.05) in demographics, comorbidities, laboratory values (including serum albumin, ESR, CRP, etc), or surgical parameters (approach, anesthesia, operative time, etc), except for a clinically negligible serum sodium difference (*P* < .05) within normal ranges that did not affect model outcomes.

**Table 1:** Comparison of baseline characteristics between training and validation cohorts.

Variables	Total (n = 419)	Test (n = 126)	Train (n = 293)	Statistic	*P*
Weight (kg)	58.09 ± 10.47	57.94 ± 11.85	58.15 ± 9.84	*t* = −0.19	.848
Height (cm)	158.62 ± 8.82	158.95 ± 9.22	158.48 ± 8.65	*t* = 0.50	.616
Operative time (min)	104.51 ± 31.06	105.97 ± 31.31	103.88 ± 30.98	*t* = 0.63	.528
Incision length (cm)	16.05 ± 2.91	16.24 ± 3.13	15.97 ± 2.81	*t* = 0.87	.386
WBC (×10⁹/L)	7.20 ± 2.46	6.90 ± 2.07	7.33 ± 2.60	*t* = −1.64	.101
Hemoglobin (g/L)	127.92 ± 18.32	128.23 ± 19.46	127.79 ± 17.83	*t* = 0.22	.823
HDL-C (mmol/L)	1.31 ± 0.38	1.28 ± 0.36	1.32 ± 0.39	*t* = −0.98	.327
LDL-C (mmol/L)	2.85 ± 0.84	2.80 ± 0.79	2.87 ± 0.86	*t* = −0.84	.401
ALP (U/L)	92.92 ± 33.67	92.33 ± 35.37	93.17 ± 32.97	*t* = −0.24	.813
Sodium (mmol/L)	142.69 ± 2.61	142.28 ± 2.74	142.86 ± 2.53	*t* = −2.10	.036
Calcium (mmol/L)	2.32 ± 0.12	2.32 ± 0.13	2.32 ± 0.12	*t* = −0.05	.964
Albumin (g/L)	39.47 ± 3.93	39.49 ± 4.52	39.46 ± 3.66	*t* = 0.07	.942
Globulin (g/L)	31.91 ± 4.95	31.71 ± 5.44	31.99 ± 4.73	*t* = −0.52	.605
Total bilirubin (μmol/L)	12.19 ± 5.71	12.59 ± 6.27	12.02 ± 5.45	*t* = 0.93	.351
Uric acid (μmol/L)	355.17 ± 106.19	349.60 ± 104.24	357.56 ± 107.11	*t* = −0.70	.482
Blood loss (mL)	100.00 (100.00, 150.00)	100.00 (100.00, 150.00)	100.00 (100.00, 150.00)	*Z* = −0.39	.694
Drainage (mL)	0.00 (0.00, 125.00)	0.00 (0.00, 135.00)	0.00 (0.00, 121.00)	*Z* = −0.33	.745
ESR (mm/h)	25.00 (14.00, 40.00)	23.00 (11.00, 40.00)	25.00 (15.00, 39.00)	*Z* = −0.59	.556
CRP (mg/L)	0.68 (0.28, 1.79)	0.64 (0.25, 1.51)	0.71 (0.28, 1.99)	*Z* = −0.82	.411
Triglycerides (mmol/L)	1.18 (0.88, 1.64)	1.21 (0.90, 1.65)	1.18 (0.87, 1.63)	*Z* = −0.32	.748
Gender				χ² = 0.90	.343
Male	208 (49.64)	67 (53.17)	141 (48.12)		
Female	211 (50.36)	59 (46.83)	152 (51.88)		
Age				χ² = 2.09	.148
<65 yr	237 (56.56)	78 (61.90)	159 (54.27)		
≥65 yr	182 (43.44)	48 (38.10)	134 (45.73)		
Anesthesia				χ² = 1.38	.240
CSEA	367 (87.59)	114 (90.48)	253 (86.35)		
GA	52 (12.41)	12 (9.52)	40 (13.65)		
Surgical approach				χ² = 0.53	.466
Posterolateral	403 (96.18)	123 (97.62)	280 (95.56)		
Direct anterior	16 (3.82)	3 (2.38)	13 (4.44)		
Drain placement				χ² = 0.32	.571
No	244 (58.23)	76 (60.32)	168 (57.34)		
Yes	175 (41.77)	50 (39.68)	125 (42.66)		
Osteoporosis				χ² = 1.05	.304
No	125 (29.83)	42 (33.33)	83 (28.33)		
Yes	294 (70.17)	84 (66.67)	210 (71.67)		
Hypertension				χ² = 3.04	.081
No	305 (72.79)	99 (78.57)	206 (70.31)		
Yes	114 (27.21)	27 (21.43)	87 (29.69)		
Diabetes				χ² = 3.37	.067
No	383 (91.41)	120 (95.24)	263 (89.76)		
Yes	36 (8.59)	6 (4.76)	30 (10.24)		
COPD				χ² = 0.00	.976
No	386 (92.12)	116 (92.06)	270 (92.15)		
Yes	33 (7.88)	10 (7.94)	23 (7.85)		
Chronic gastritis				χ² = 2.19	.139
No	406 (96.90)	125 (99.21)	281 (95.90)		
Yes	13 (3.10)	1 (0.79)	12 (4.10)		

ALP = alkaline phosphatase, COPD = chronic obstructive pulmonary disease, CRP = C-reactive protein, ESR = erythrocyte sedimentation rate, HDL-C = high-density lipoprotein cholesterol, LDL-C = low-density lipoprotein cholesterol, WBC = white blood cell.

### 3.2. Multicollinearity testing

As shown in Table 2, all variables had tolerance values >0.1 and variance inflation factor <5 (maximum: 3.56), indicating no multicollinearity issues – all independent variables were mutually independent or showed very low correlations.

**Table 2:** Multicollinearity assessment results.

Variable	Tolerance	VIF	Variable	Tolerance	VIF
Sex	0.430	2.324	Chronic gastritis	0.922	1.085
Age	0.781	1.281	White blood cell count	0.763	1.311
Weight	0.509	1.966	Hemoglobin	0.500	2.002
Height	0.463	2.161	Erythrocyte sedimentation rate	0.392	2.553
Operative time	0.647	1.545	C-reactive protein	0.597	1.675
Anesthesia method	0.744	1.344	Triglycerides	0.735	1.361
Surgical approach	0.540	1.853	High-density lipoprotein	0.735	1.361
Incision length	0.515	1.943	Low-density lipoprotein	0.808	1.237
Blood loss	0.663	1.507	Alkaline phosphatase	0.863	1.159
Drain placement	0.271	3.688	Serum sodium	0.843	1.187
Drainage volume	0.359	2.784	Serum calcium	0.553	1.807
Osteoporosis	0.732	1.366	Serum albumin	0.531	1.884
Hypertension history	0.827	1.209	Globulin	0.469	2.132
Diabetes history	0.858	1.166	Total bilirubin	0.810	1.234
COPD history	0.901	1.110	Uric acid	0.678	1.476

COPD = chronic obstructive pulmonary disease, VIF = variance inflation factor.

### 3.3. Univariate and multivariate logistic regression analysis in the training cohort

In the univariate logistic regression analysis of the training cohort, variables with *P* < .05 were considered significantly associated with POHA. A total of 11 significant predictors were identified (Table 3), including sex, age, operative time, blood loss, hemoglobin, ESR, CRP, low-density lipoprotein, alkaline phosphatase (ALP), serum albumin, and globulin. These 11 variables were included in the multivariate logistic regression analysis (*P* < .05 indicating statistical significance), which revealed that sex, operative time, ESR, ALP, and serum albumin were independent risk factors for POHA after THA (Table 4).

**Table 3:** Univariate logistic regression analysis.

Variables	β	SE	*Z*	*P*-value	OR (95% CI)
Sex					
Male	–	–	–	Ref	1.00 (Reference)
Female	0.82	0.32	2.54	.011	2.26 (1.21–4.25)
Age					
<65 yr	–	–	–	Ref	1.00 (Reference)
>65yr	0.63	0.31	2.04	.041	1.87 (1.03–3.43)
Anesthesia					
CSEA	–	–	–	Ref	1.00 (Reference)
General	0.14	0.43	0.34	.736	1.16 (0.50–2.67)
Surgical approach					
Posterolateral	–	–	–	Ref	1.00 (Reference)
Direct anterior	−1.01	1.05	−0.96	.339	0.37 (0.05–2.87)
Drainage					
No	–	–	–	Ref	1.00 (Reference)
Yes	−0.25	0.31	−0.80	.424	0.78 (0.42–1.44)
Osteoporosis					
No	–	–	–	Ref	1.00 (Reference)
Yes	0.24	0.35	0.68	.498	1.27 (0.64–2.51)
Hypertension					
No	–	–	–	Ref	1.00 (Reference)
Yes	0.14	0.33	0.42	.675	1.15 (0.60–2.18)
Diabetes					
No	–	–	–	Ref	1.00 (Reference)
Yes	0.14	0.48	0.29	.774	1.15 (0.44–2.97)
COPD					
No	–	–	–	Ref	1.00 (Reference)
Yes	−0.05	0.57	−0.09	.928	0.95 (0.31–2.92)
Chronic gastritis					
No	–	–	–	Ref	1.00 (Reference)
Yes	0.43	0.68	0.63	.528	1.54 (0.40–5.89)
Weight	−0.02	0.02	−1.37	.171	0.98 (0.95–1.01)
Height	−0.02	0.02	−1.41	.158	0.98 (0.94–1.01)
Operative time	0.01	0.00	2.28	.023	1.01 (1.01–1.02)
Incision length	0.05	0.05	0.90	.368	1.05 (0.94–1.17)
Blood loss	0.01	0.00	2.06	.039	1.01 (1.01–1.01)
Drainage volume	−0.00	0.00	−0.46	.643	1.00 (1.00–1.00)
WBC	−0.06	0.06	−1.01	.312	0.94 (0.83–1.06)
ESR	0.04	0.01	5.21	<.001	1.04 (1.02–1.06)
CRP	0.14	0.04	3.45	<.001	1.15 (1.06–1.24)
Triglycerides	−0.33	0.23	−1.44	.150	0.72 (0.45–1.13)
Hemoglobin	−0.04	0.01	−4.71	<.001	0.96 (0.94–0.98)
HDL	−0.49	0.41	−1.19	.233	0.61 (0.27–1.37)
LDL	−0.42	0.19	−2.21	.027	0.66 (0.45–0.95)
ALP	0.01	0.00	2.53	.011	1.01 (1.01–1.02)
Serum sodium	0.00	0.06	0.07	.941	1.00 (0.89–1.13)
Serum calcium	−0.58	1.31	−0.45	.656	0.56 (0.04–7.32)
Serum albumin	−0.25	0.05	−5.12	<.001	0.78 (0.71–0.86)
Globulin	0.11	0.03	3.22	.001	1.11 (1.04–1.19)
Total bilirubin	−0.04	0.03	−1.45	.147	0.96 (0.90–1.02)
Uric acid	−0.00	0.00	−1.08	.279	1.00 (1.00–1.00)

ALP = alkaline phosphatase, CI = confidence interval, COPD = chronic obstructive pulmonary disease, CRP = C-reactive protein, ESR = erythrocyte sedimentation rate, HDL-C = high-density lipoprotein cholesterol, LDL-C = low-density lipoprotein cholesterol, OR = odds ratio, SE = standard error.

**Table 4:** Multivariate logistic regression analysis.

Variables	β	SE	*Z*	*P*-value	OR (95% CI)
Sex	–	–	–	–	–
Male	–	–	–	Ref	1.00 (Reference)
Female	1.01	0.38	2.68	.007	2.74 (1.31–5.72)
Operative time	0.02	0.01	2.73	.006	1.02 (1.01–1.03)
ESR	0.03	0.01	3.32	<.001	1.03 (1.01–1.05)
ALP	0.01	0.01	2.01	.045	1.01 (1.01–1.02)
Serum albumin	−0.23	0.05	−4.32	<.001	0.80 (0.72–0.88)

ALP = alkaline phosphatase, CI = confidence interval, ESR = erythrocyte sedimentation rate, OR = odds ratio, SE = standard error.

### 3.4. Development and validation of the predictive model

**Figure 1.**
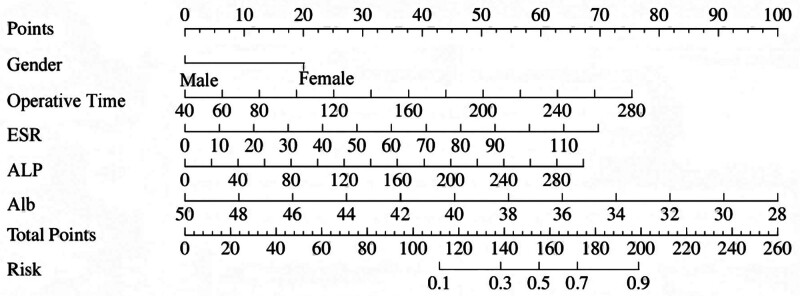
Risk prediction nomogram (derivation cohort). ALP = alkaline phosphatase, ESR = erythrocyte sedimentation rate.

Based on the multivariate logistic regression analysis results, the 5 identified risk factors were incorporated as predictors to construct a nomogram for predicting POHA after THA using the training cohort (see Fig. 1). In clinical practice, physicians can utilize this nomogram by assigning points to each risk factor, summing the total points, and correlating the total score with the corresponding risk probability of POHA.

### 3.5. Evaluation of the predictive model

**Figure 2.**
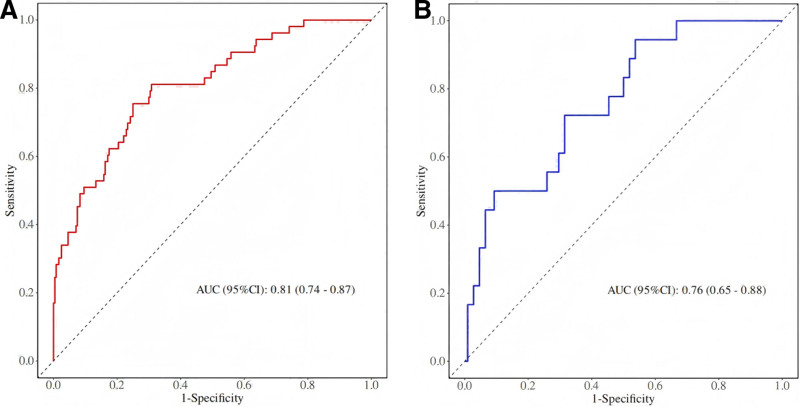
(A) ROC curve of the training cohort; (B) ROC curve of the validation cohort. AUC = area under the curve, CI = confidence interval, ROC = receiver operating characteristic.

**Figure 3.**
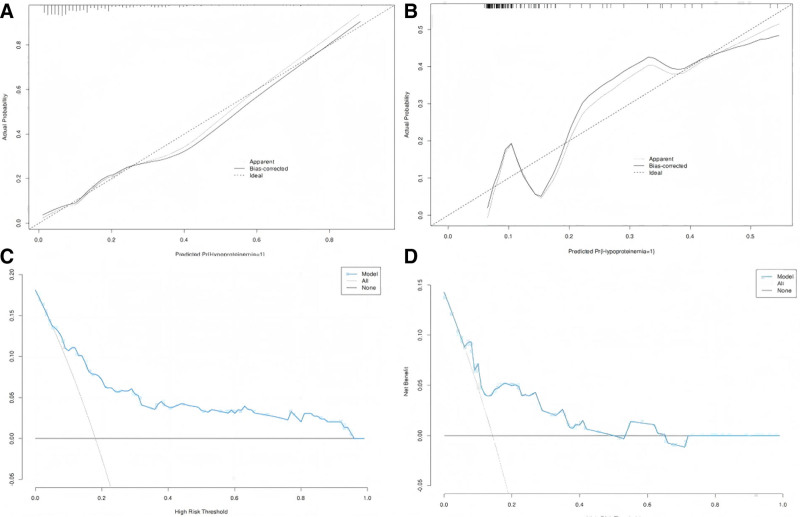
(A) Calibration curve of the training cohort; (B) calibration curve of the validation cohort; (C) decision curve analysis of the training cohort; and (D) decision curve analysis of the validation cohort.

The AUC values for the training and validation cohorts were 0.81 (95% CI: 0.74–0.87) and 0.76 (95% CI: 0.65–0.88), respectively, indicating high accuracy in predicting POHA risk after THA (Fig. 2A, B). The difference in AUC between the 2 groups was ≤0.5, and the Hosmer–Lemeshow test yielded *P*-values of .27 (training) and 0.34 (validation), suggesting a low risk of overfitting. The calibration curves demonstrated excellent agreement between predicted and observed outcomes in both cohorts (Fig. 3A, B), confirming the model’s reliable discriminative ability and calibration performance. Decision curve analysis revealed greater net benefits across risk thresholds of 0.05 to 0.96 (training) and 0.06 to 0.65 (validation) for predicting POHA (Fig. 3C, D).

## 4. Discussion

This study found that the overall incidence of POHA after THA was 16.90%. It is important to clarify that POHA is not equivalent to chronic HA, as it primarily results from the combined effects of inflammatory responses, surgical trauma, and other factors, manifesting as a short-term but dramatic fluctuation in serum albumin levels. This study excluded patients with conditions likely to cause chronic HA, such as multiple fractures, malignancies, severe digestive diseases, cirrhosis, and uremia, focusing instead on general patients and perioperative factors.

In our study, female sex was identified as an independent risk factor for POHA. Research by Rondanelli on lipid profiles and sex/BMI-related differences demonstrated that women have significantly higher levels of arachidonic acid compared to men, indicating notable differences in humoral and cell-mediated immune responses between sexes.^[15]^ Arachidonic acid has potent pro-inflammatory effects, leading to stronger inflammatory and innate immune responses in women following bacterial/viral infections or vaccinations.^[16]^ Unfortunately, this pattern also predisposes women to higher rates of inflammatory and autoimmune diseases.^[17]^ The primary mechanism of POHA involves inflammatory mediators increasing endothelial cell gap permeability or directly damaging vascular endothelial cells, ultimately causing albumin leakage. The pro-inflammatory tendency in women may exacerbate this process.^[18]^

There are significant differences in body composition between males and females. Studies demonstrate that females have proportionally higher fat mass, while males possess greater muscle mass.^[19]^ This divergence creates 2 potential pathways for increased POHA risk in females: First, adipose tissue not only serves energy storage but also exhibits endocrine functions, secreting pro-inflammatory cytokines including TNF-α, IL-6, and IL-8.^[20]^ The hip region involved in THA contains abundant adipose tissue. Surgical incisions may trigger and amplify inflammatory responses, where chemokines, inflammatory cytokines, and adipokines drive immune cell infiltration into adipose tissue, promoting inflammation initiation and progression within fat compartments.^[21]^ This mechanism is corroborated by findings of serum albumin accumulation at surgical wound sites.^[22]^ Second, as the body’s largest protein reservoir, skeletal muscle is critical for maintaining nitrogen balance and physiological stress responses. Females’ lower muscle mass implies reduced protein reserves, which limits proteolytic capacity during trauma or postoperative recovery, consequently decreasing amino acid release (the substrates for protein synthesis).^[23]^

Our study identified low preoperative serum albumin as a POHA risk factor. Human serum albumin (66.5 kDa, negatively charged, elliptical) constitutes 50% of plasma proteins, with a half-life of 17 to 19 days.^[24]^ Normally, albumin synthesis occurs in only 20% to 30% of hepatocytes, with no storage capacity. After synthesis, albumin circulates between intravascular and interstitial spaces.^[25]^ Thus, low preoperative albumin reflects an inadequate short-term reserve.

Additionally, HA impairs secondary albumin functions: drug/nutrient transport, capillary integrity maintenance, and antioxidant activity.^[26]^ Reduced inflammatory clearance and vascular protection may exacerbate surgical trauma. While albumin is a flawed nutritional marker in acute illness, it remains a surrogate for nutritional status.^[27]^ Poor nutrition may indicate impaired hepatic synthesis or underlying protein-deficient conditions.^[28]^

Elevated ESR and ALP were also risk factors. High ESR reflects systemic inflammation and HA, as albumin inhibits erythrocyte aggregation.^[29]^ Conversely, inflammatory cytokines (e.g., TNF-α, IL-6) suppress hepatic protein synthesis^[30]^ and increase vascular permeability, accelerating albumin extravasation.^[31]^ ALP, beyond its role in liver/bone metabolism, mediates pro-inflammatory cytokines (e.g., CRP, IL-6) in chronic inflammation.^[32,33]^ Elevated ALP may also indicate subclinical muscle metabolic dysfunction, exacerbated by surgical stress, impairing protein synthesis for tissue repair.^[34,35]^ Combined, these factors increase POHA risk.

Prolonged operative time was another risk factor. Mommsen et al found that extended surgery elevates stress hormones, disrupting protein metabolism, inflammation, and immune function via a trauma-stress network,^[36]^ leading to albumin loss. Longer procedures also correlate with greater blood loss (overt or hidden), directly reducing intravascular albumin.^[37]^

This study has several limitations. First, the surgical procedures were not performed by a single surgeon, which may introduce potential bias. Second, as a retrospective study, selection bias may exist in data collection and feature selection, necessitating prospective studies to validate the reliability of the results. Finally, being a single-center study, the representativeness and generalizability of the sample may be limited, and future multicenter, large-sample studies are needed to enhance the external validity of the conclusions.

## 5. Conclusion

Female sex, prolonged operative time, high ESR, and high ALP are risk factors for POHA after THA, while high preoperative serum albumin is protective. The nomogram incorporating these factors can clinically predict POHA risk. Orthopedic surgeons should optimize perioperative management by addressing POHA and its determinants to reduce its incidence.

## Author contributions

**Conceptualization:** Yongshuo Li, Wengang Zhu.

**Data curation:** Zhongyou Yu, Yilv Zhang, Nanhai Qiu.

**Investigation:** Yilv Zhang, Nanhai Qiu.

**Supervision:** Yongshuo Li, Wengang Zhu.

**Visualization:** Zhongyou Yu.

**Writing** – **original draft:** Yongshuo Li.

**Writing** – **review & editing:** Zhongyou Yu, Yongshuo Li, Yilv Zhang, Nanhai Qiu, Wengang Zhu.
